# Multisectoral Policies and Programming: High-Income Countries Can and Should Be Learning From the Philippines and Other Low- and Middle-Income Countries

**DOI:** 10.9745/GHSP-D-21-00541

**Published:** 2021-09-30

**Authors:** Madeleine Short Fabic

**Affiliations:** a United States Agency for International Development, Washington, DC, USA.

## Abstract

The global health field will miss key learning opportunities if it continues to make a false distinction between research of relevance to lowand middle-income countries and research of relevance to high-income countries.

See related article by Siy Van et al.

The global health field often makes a false distinction between research of relevance to low- and middle-income countries (LMICs) and research of relevance to high-income countries (HICs). This practice feeds into and mirrors development practices wherein technical assistance unidirectionally flows from HICs to LMICs; where HICs have experience and teachings to share with LMICs but generally do not recognize the reverse is also true; and where partnership is limited by power hierarchies that elevate the expertise and knowledge of HICs above that of LMICs.^1^

Reading the Siy Van et al. article,^2^ “Trends in national-level governance and implementation of the Philippines’ Responsible Parenthood and Reproductive Health Law from 2014 to 2020,” reminded me of the opportunities we miss if the global health field implicitly insists that its research and programmatic learnings are of primary relevance to LMICs. Indeed, I would argue that the challenges the authors present related to multisectoral coordination, collaboration, integration, and accountability parallel issues of public health importance in my home country, the United States. Through this lens, I offer a brief synthesis of the challenges described by Siy Van et al.,^2^ as well as some thoughts on how learnings from the Philippines’ experience could be applied to the United States. In the U.S. context, I focus on 2 multisectoral approaches to pandemic prevention, detection, and response that have increased in prominence in the wake of the coronavirus disease (COVID-19) pandemic—global health security and One Health.^3^^,^^4^

## Problem A: Siloed Implementation Hampered Success in Attaining Objectives and Goals

### Findings

Governmental collaboration floundered despite the 2012’s Responsible Parenthood and Reproductive Health Act (RPRH)^5^ law’s guiding principles, which call for “a multifaceted process” that necessitates

*the harmonization and integration of policies, plans, programs and projects that seek to uplift the quality of life of the people.*

Governmental agencies more readily fulfilled mandates that did not require coordination either within or between agencies. Conversely, agencies delayed or altogether missed mandates that relied on multiple institutional structures for reasons generally attributed to bureaucratic delays and inefficiencies. The RPRH was specific about the role of the Department of Health but lacked specificity about the role of other governmental agencies. Additionally, the RPRH law outlined a host of issues to be addressed but did not identify how a multisectoral approach would better address the issues than a set of siloed approaches.

### Recommendations

Competing sectoral interests can be partially mitigated through legislation. As the U.S. Congress contemplates legislation on the heels of the current pandemic, multisectoral governance, including “One Health” and global health security, seems to be gaining traction.^*^ The Philippines’ experience provides valuable lessons for new legislation: to successfully address multisectoral problems, legislation should clearly articulate a problem statement that identifies why a multisectoral response is required and preferable to a siloed response; additionally, legislation must clearly articulate the roles and responsibilities of each identified organizational entity. Threading this legislative needle of being directive without being overly prescriptive will be an ongoing challenge. Executive branch agencies can help alleviate this challenge by making clear via strategy documents and in Congressional briefings how each agency understands the multisectoral problem, what unique capabilities each agency brings to bear, and how each agency perceives the opportunities afforded by holistic policy making.

## Problem B: Dominance of 1 Technical Area Over All Others Overly Limited the Scope of Work

### Findings

Despite the multidimensional nature of reproductive health and the fact that the RPRH law defined reproductive health care as having 12 elements, RPRH implementers limited the scope of their efforts, focusing predominantly on family planning. As Siy Van et al.^2^ report, family planning received “disproportionately more efforts and resources from RPRH implementers than did any other element.” This may have occurred in part because the National Implementation Team, created in 2014 to manage and coordinate interagency RPRH activities, chose key result areas that included only 5 of the 12 elements. Additionally, family planning programs had a strong foundation, having already been explicitly required by law, with widespread implementation throughout the country. Family planning program implementation challenges were already well known. Meanwhile, family planning was under continued attack by various entities in the Philippines, prompting its proponents to amplify focus and attention. Ultimately, interagency conversations focused narrowly on family planning commodities and service provision to the detriment of focus on other elements of reproductive health.

### Recommendations

The adage, “what you measure is what you get,” is worth reciting. If multisectoral programs are intended to have multiple, multisectoral impacts, then legislative and executive branches of government must identify and incorporate multisectoral indicators into monitoring and evaluation plans, ideally in collaboration with civil society and other nongovernmental stakeholders. In the context of One Health, this means that animal, human, and environmental health should all have equal footing and focus, including measurement focus. It also means that no one sector can dominate the narrative or dominate the policy and programming approach. In the Philippines, for example, the Department of Health was tasked with leading the overall reproductive health effort, and the approach became dominated by the Department’s biomedical understanding of the problem. As the U.S. Government and new administration consider how best to organize to tackle complex problems like pandemic prevention or climate change, organizational structures that elevate multiple sets of expertise and problem perspectives are more likely to yield effective multisectoral responses. For example, were the United States to support a pandemic prevention convening body, rotating organizational leadership chairs from varied executive branch agencies with stewardship and support from the White House and/or Congress can serve to elevate multiple perspectives in advancement of an overarching strategy. Additionally, moving away from narratives like “global health security,” where a human health context dominates the discourse and programming, and toward narratives like “One Health,” where an interconnected human-animal-environment health context dominates, can further support effective multisectoral collaboration and programming approaches.

## Problem C: Focus on Programmatic Concerns Limited Development and Implementation of an Overarching Multisectoral Strategy and Response, Which Inhibited Success

### Findings

Interagency meetings narrowly focused on programmatic issues that concerned only 1 or 2 agencies, never allowing time or space for collaborations to coalesce. Such narrow emphasis likely stemmed from a lack of clear multisectoral expectations and identified outputs. Moreover, accountability tools were missing. Without an objective arbiter, interagency disagreements were rarely raised or addressed. And without a shared monitoring and evaluation plan that included measures of multisectoral success, indicators of progress remained within their original organizational silos.

### Recommendations

Multisectoral work requires effective stewardship and leadership, which Siy Van et al. suggest needs 3 things: (1) clear outcomes and goals known by all engaged parties; (2) necessary resources—including financial, human, policy infrastructure, and related tools to enforce agreed upon mandates/deliverables; and (3) designated participants with clearly identified roles and responsibilities. While leadership within U.S. agencies and large bureaucracies is generally vertical and top-down, horizontal stewardship and leadership can also play a role in forging relationships and partnerships, building trust, and identifying shared goals. Learning from the Philippines’ experience, were the United States to move toward multisectoral One Health work, it would benefit by having top-down leadership from the Executive branch, for example, a Presidential Czar, coupled with horizontal leadership from various engaged agencies. The legislative branch would need to ensure that the efforts were appropriately financially resourced, and each respective agency would need to ensure that requisite staff were identified and actively engaged. At the outset, a multisectoral group would need to develop shared, codified outcomes and goals, and a monitoring and evaluation plan that effectively represents multisectoral work, which is not simply a repack-aging of existing workstreams but is instead a new way of collaboratively operating.

In conclusion, and as I hope I have well illustrated herein, the United States and other HICs can and should be learning from the experiences of the Philippines and other LMICs.

## Footnotes

* A quick search on July 19, 2021, of Congress.gov reveals that since the start of the current 2021–2022 U.S. Congress, 10 pieces of legislation have been introduced in the House or Senate that include the term “One Health,” and 21 pieces of legislation have been introduced in the House or Senate that include the term “global health security.”

## Notes

**First published online:** September 8, 2021.
